# Accidental Coverage of Both Renal Arteries during Infrarenal Aortic Stent-Graft Implantation: Cause and Treatment

**DOI:** 10.1155/2014/710742

**Published:** 2014-12-03

**Authors:** Umberto Marcello Bracale, Anna Maria Giribono, Gaetano Vitale, Donatella Narese, Gianpaolo Santini, Luca del Guercio

**Affiliations:** ^1^Department of Vascular and Endovascular Surgery, University Federico II of Naples, Naples, Italy; ^2^Department of Diagnostic Imaging, Section of General and Emergency Radiology, Cardarelli Hospital of Naples, 80131 Naples, Italy

## Abstract

The purpose of this paper is to report a salvage maneuver for accidental coverage of both renal arteries during endovascular aneurysm repair (EVAR) of an infrarenal abdominal aortic aneurysm (AAA). A 72-year-old female with a 6 cm infrarenal abdominal aortic aneurysm was treated by endovascular means with a standard bifurcated graft. Upon completing an angiogram, both renal arteries were found to be accidentally occluded. Through a left percutaneous brachial approach, the right renal artery was catheterized and a chimney stent was deployed; however this was not possible for the left renal artery. A retroperitoneal surgical approach was therefore carried out with a retrograde chimney stent implanted to restore blood flow. After three months, both renal arteries were patent and renal function was not different from the baseline. Both endovascular with percutaneous access via the brachial artery and open retroperitoneal approaches with retrograde catheterization are feasible rescue techniques to recanalize the accidentally occluded renal arteries during EVAR.

## 1. Introduction

Unintentional coverage of both renal arteries after endovascular aneurysm repair (EVAR) for abdominal aortic aneurysm (AAA) remains one of the most undesirable complications for the vascular surgeon. Several endovascular techniques found in the literature describe adjusting the malpositioned stent-graft or revascularizing the occluded artery [1–3]; however, to date, no consensus exists as to the best approach to resolving this occurrence. Herein we report on accidental ostial coverage of both renal arteries during EVAR and present an alternate, combined open/endovascular approach used to salvage one of the occluded renal arteries.

## 2. Case Report

**Figure 1:**
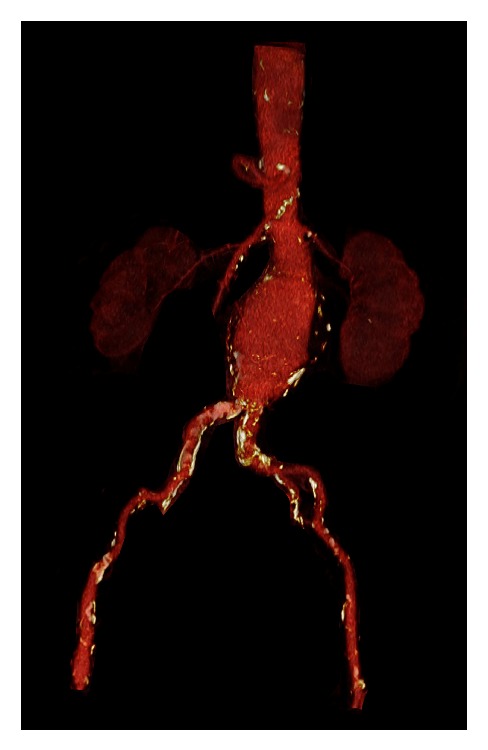
Preoperative angio-CT scan showing a 6 cm abdominal aortic aneurysm suitable for EVAR.

A 72-year-old female with a 6 cm diameter asymptomatic infrarenal abdominal aortic aneurysm as revealed by previous computed tomography angiography (CTA) measurements (Figure 1) was evaluated for standard EVAR. The patient's clinical history was remarkable for hypertension, hyperlipidemia, and low renal insufficiency (creatinine level of 1.2 mg/dL and BUN 72 mg/dL).

**Figure 2:**
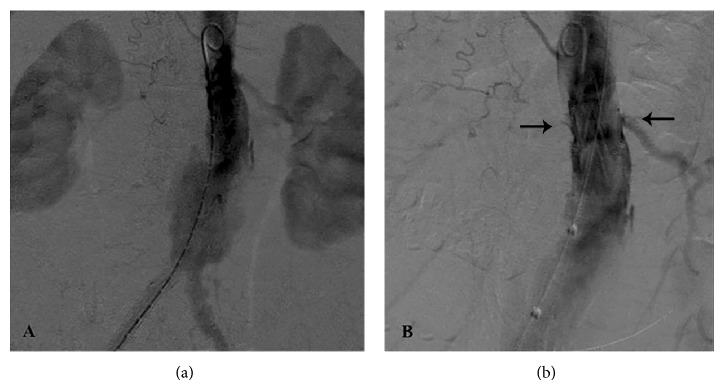
Completion angiogram after stent-graft deployment showing type 1a endoleak (a). After aortic cuff extension placement, both renal arteries were covered (arrows) (b).

**Figure 3:**
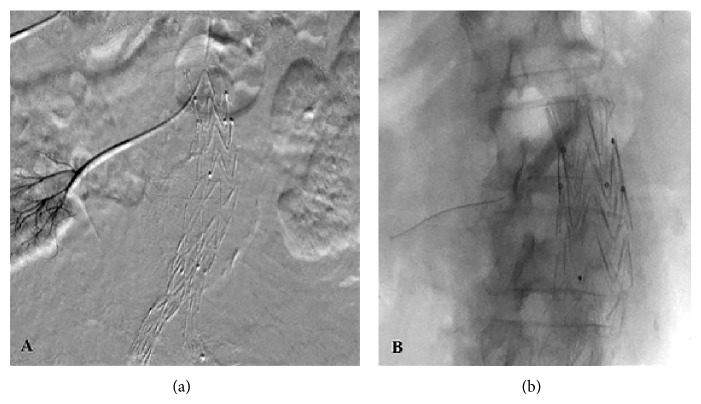
Successful recanalization (a) and stent placement (b) into right renal artery from left brachial approach.

**Figure 4:**
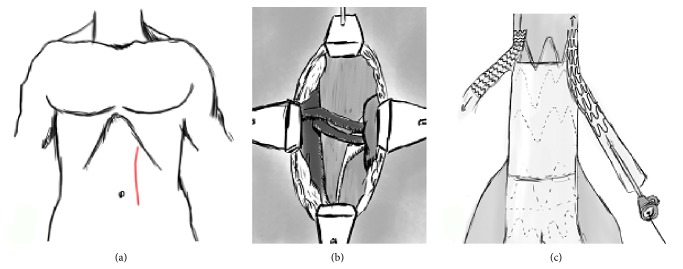
Left retroperitoneal approach: abdominal incision for left retroperitoneal approach to the renal artery (a). Surgical exposure of the main trunk of the left renal artery (b). Retrograde insertion of a 5 Fr short sheath and subsequent 6 mm × 18 mm balloon expandable stent deployment (c).

**Figure 5:**
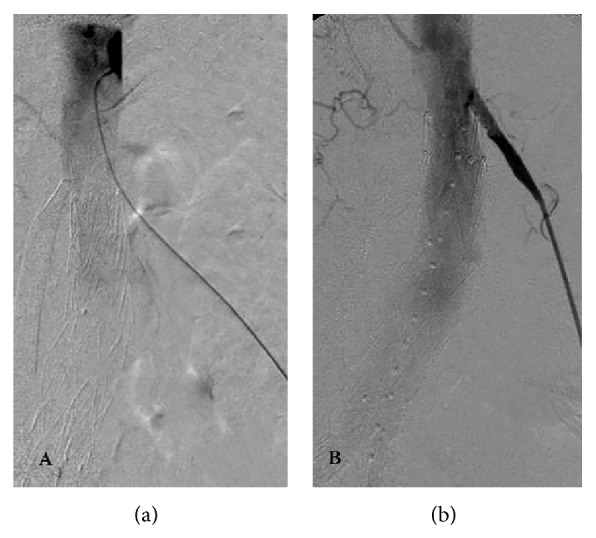
Retrograde cannulation of the left renal artery with a 4 Fr JR catheter; injection of contrast medium through the catheter confirming the successful reentering into the aorta (a). Completion angiogram after retrograde bare metal stent placement confirming patency of the renal artery (b).

A 28 mm × 14 mm E-vita abdominal stent-graft (JOTEC, Hechingen, Germany) was selected for repairing the aneurysm. In the operating theatre, the patient was put under general anesthesia at her choice and both common femoral arteries were surgically exposed. The stent-graft was deployed and the completion angiogram showed a type 1a endoleak, which was treated with a proximal aortic extension cuff (Figure 2(a)). Insertion and deployment of the aortic cuff system proved tricky with much friction due, perhaps, to severe angulation of the calcified iliac vessels and the presence of the previously implanted prosthesis. A subsequent angiogram revealed coverage of both renal arteries (Figure 2(b)). Selective catheterization of the right renal artery was achieved through left percutaneous brachial access and a 6 mm × 18 mm balloon-expandable stent (Express SD, Boston Scientific, Natick, MA, USA) deployed in a “chimney” fashion (Figures 3(a) and 3(b)). Attempts to catheterize the left renal artery failed requiring an open exposure through a left retroperitoneal surgical approach (Figures 4(a), 4(b), and 4(c)). A retrograde puncture of the occluded renal artery was carried out and a 5 Fr sheath positioned in. With a 0.035′′ wire (Zip Guidewire, Boston Scientific, Natick, MA, USA) and a JR 4 Fr catheter (Cordis, Johnson and Johnson, Miami, USA) retrograde catheterization of the occluded renal artery was performed (Figure 5(a)) and another 6 mm × 18 mm balloon-expandable stent (Express SD, Boston Scientific, Natick, MA, USA) was implanted (Figure 5(b)). Completion angiogram confirmed patency of both renal arteries stents. The puncture hole in the renal artery was closed with prolene 6-0 (Ethicon Ltd., Edinburg, UK). Total operative time of both procedures was 310 min and blood loss was 700 mL. Fluoroscopy time was 98 min and total contrast volume used was 380 mL (Visipaque 270 mg/mL, GE Healthcare B.V., Eindhoven, The Netherlands).

**Figure 6:**
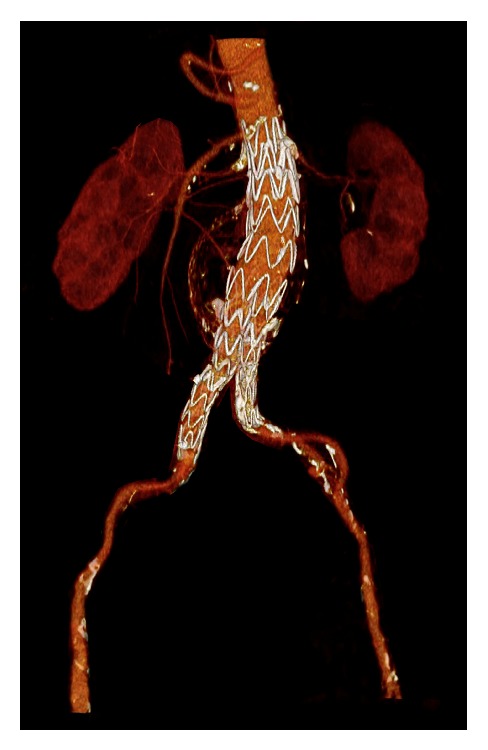
The angio-CT scan at the 3-month follow-up showing patency of renal stents and nice perfusion of both kidneys with no evidence of any endoleak.

After 24 hours a transient decline of renal function was noted with an increase of serum creatinine (2.1 mg/dL). The patient's postoperative course was uneventful and renal function returned to baseline within four days (creatinine 1.2 mg/dL). The patient was discharged home on postoperative day 8 and at three months both duplex examination and CT scan revealed patency of renal arteries with no evidence of any types of endoleaks nor recurrent migration of any part of the stent-graft material (Figure 6) and stable serum creatinine.

## 3. Discussion

Inadvertent occlusion of one or both renal arteries to treat infrarenal AAA following stent-graft implantation is one of the severest EVAR complications and its treatment strategy, particularly in large trials, is not well documented leading to the currently unknown incidence rate. Though this phenomenon is extremely rare, if not identified and treated early, acute tubular necrosis and permanent renal failure requiring hemodialysis can occur.

Katzen et al. first described the case of a patient who underwent infrarenal stent-graft procedure and postoperatively developed dialysis-dependent renal failure due to a retrograde migration of the device, which occluded the patient's bilateral renal arteries. Considering the patient's comorbidities and the length of time since the kidneys had been perfused, the decision was made to continue with dialysis and leave the stent-graft in place [4]. Several techniques are being found in the current published literature that attempt to avoid such an undesirable complication. In 2010 Hamish et al. published a literature review and the results of a questionnaire on the incidence and management of both renal artery occlusions following stent-graft malpositioning. The questionnaire was sent to all listed members of the Vascular Society of Great Britain and Ireland. Forty percent (27/68) of the respondents had almost experienced a case of bilateral renal artery occlusion during EVAR. Two-thirds (67%, 18/27) of the surgeons stated a preference for revascularizing the kidneys endovascularly, 7 preferred to convert to open repair, and 1 surgeon favored iliorenal bypass while another suggested splenorenal bypass. Following intervention, 15 (56%) out of 27 surgeons reported achieving revascularization resulting in a return to baseline serum creatinine, 7 (26%) achieved partial recovery of the patient's serum creatinine, 3 (11%) had a patient on permanent dialysis, and 2 (7%) had patients who had died (following open repair and endovascular procedure, resp.) [5]. More recently, Adu et al. [1] present a good overview of all endovascular and surgical procedures for renal artery salvage following unintentional coverage. Amongst these, the antegrade renal artery chimney stent, also described by Inan et al. [3], is considered a well-documented and effective endovascular rescue technique; however its feasibility depends on a leak or some space between the aorta and the main body of the stent-graft in order to advance a guidewire into the renal artery.

Such was the case of the right renal artery revascularization we performed on our patient, which was cannulated through left brachial access, a preferred route in most cases. Endovascular techniques provide an effective strategy and should be used as the initial therapy to restoring renal perfusion and salvage renal function, while open surgical bypass should be considered in those patients for whom endovascular therapy has failed [6]. Typically, risk of failure increases with a difficult anatomy, such as an angulation of the iliac vessels and aortic neck or sharp take-off of the renal arteries. Hamish et al. [7] reported a case of a successful hepatorenal bypass performed on the right kidney using the origin of the gastroduodenal artery as inflow while the left kidney was revascularized through a splenorenal bypass. Procedures such as these, as well as open conversion, are technically demanding and require large vessel dissection and/or supraceliac aortic clamping.

Alternately, retrograde access catheterization, though a less invasive retroperitoneal open approach, is a relatively easy technique allowing for assessment of renal ischemia and permitting full control of distal outflow to prevent embolization. A similar technique was adopted by Vourliotakis et al. [8] to reopen an accidentally crushed covered stent during implantation of a fenestrated stent-graft. As with other endovascular procedures, certain limitations related to the stenting of renal arteries exist, as the rate of restenosis is more common in stented arteries than in open procedures. With bypass surgery, however, in most cases it is relatively easy to retreat the target vessel with a percutaneous approach under local anesthesia and the risk of graft kinking is also avoided.

## 4. Conclusion

Combined open/endovascular technique used for renal artery salvage as an emergency maneuver for patients undergoing EVAR is a useful and an alternative technique when a surgeon finds it difficult to cannulate an occluded vessel antegradely or when bypass surgical revascularization and open conversion are not the preferred approaches.
